# Barriers and facilitators to enrollment and re-enrollment into the community health funds/Tiba Kwa Kadi (CHF/TIKA) in Tanzania: a cross-sectional inquiry on the effects of socio-demographic factors and social marketing strategies

**DOI:** 10.1186/s12913-017-2250-z

**Published:** 2017-04-27

**Authors:** Ntuli A. Kapologwe, Gibson B. Kagaruki, Albino Kalolo, Mariam Ally, Amani Shao, Manoris Meshack, Manfred Stoermer, Amena Briet, Karin Wiedenmayer, Axel Hoffman

**Affiliations:** 1 Regional Medical Office, P.O Box 320, Shinyanga, Tanzania; 2 President’s Office Regional Administration and Local Government (PORALG), P.O Box 1923, Dodoma, Tanzania; 3 National Institute for Medical Research (NIMR), Tukuyu Medical Research Center, P.O. Box 538, Tukuyu, Tanzania; 4 Department of Community Health, St. Francis University College of Health and Allied Sciences, P.O Box 175, Ifakara, Tanzania; 5 Ministry of Health, Community Development, Gender, Elderly and Children, P.O Box 9083, Dar es Salaam, Tanzania; 6 Health Promotion and System Strengthening (HPSS) Project, P.O Box 29, Dodoma, Tanzania; 7 Swiss Tropical and Public Health Institute, Socinstrasse 57, P.O. Box CH-4002, Basel, Switzerland; 8 University of Basel, Petersplatz 1, CH-4003 Basel, Switzerland

**Keywords:** Social marketing, Barriers, Facilitators, Community health funds, Tanzania

## Abstract

**Background:**

Introduction of a health insurance scheme is one of the ways to enhance access to health care services and to protect individuals from catastrophic health expenditures. Little is known on the influence of socio-demographic and social marketing strategies on enrollment and re-enrollment in the Community Health Fund/Tiba Kwa Kadi (CHF/TIKA) in Tanzania.

**Methods:**

This cross-sectional study employed quantitative methods for data collection between November 2014 and March 2015 in Singida and Shinyanga regions. Relationship between variables was obtained through Chi-square test and multivariate logistic regression.

**Results:**

We recruited 496 participants in the study. Majority (92.7%) of participants consented to participate, with 229 (49.8%) and 231 (50.2%) members and non members of CHF/TIKA respectively. Majority (90.9%) were aware of CHF/TIKA. Majority of CHF/TIKA members and non-members (90% and 68.3% respectively) reported health facility-based sensitization as the most common social marketing approach employed to market the CHF/TIKA. The most popular marketing strategies in the country including traditional dances, football games, radio, television, news papers, and mosques/church were reported by few CHF and non CHF members. Multivariate Logistic regression models revealed no significant association between social marketing strategies and enrollment, but only socio-demographics; including marital status (AOR = 2.0, 95% CI 1.1–3.8) and family size (household with ≥ 6 members) (AOR = 1.5, 95% CI 1.0–2.5), were significant factors associated with enrollment/re-enrollment rate.

**Conclusions:**

This study indicated that low level of utilization of available social marketing strategies and socio-demographic factors are the barriers for attracting members to join the schemes. There is a need for applying various social marketing strategies and considering different facilitating and impending socio-demographic factors for the growth and sustainability of the scheme as we move towards universal health coverage.

**Electronic supplementary material:**

The online version of this article (doi:10.1186/s12913-017-2250-z) contains supplementary material, which is available to authorized users.

## Background

In Africa, about half of all health care expenses are covered through out-of-pocket payments (OPP) [1]. Cost is a major barrier to those who would seek health care services and has led to inequalities in access to health care services, disenfranchising the poorer classes while pushing the middle class into financial insecurity. Introduction of a health insurance scheme is one of the ways to try the inequalities and improve access to health care services, thus, protecting the individuals from catastrophic health payments [1]. There has been some evidence that community health insurance reduces catastrophic health care expenditures and increases access to health care services [2].

The majority of African countries implement only small-scale, fragmented, community-based health insurance schemes, which only cover a city or region. The major challenge to voluntary health insurance schemes in Africa and potentially elsewhere, is the limited number of enrolled people. The majority (95%) of insurance schemes have very few (5%) enrolled members. Small-scale insurance schemes are associated with poor financial viability, due to limited risk pooling and are prone to the danger of bankruptcy [3]. Low subscription rates stem from drivers acting on the demand side, such as a lack of general knowledge about health insurance, solidarity principles, trust, and socio-cultural issues. On the other hand, the supply side factors, such as inadequate benefit packages, poor quality of health services, failure to see rationale to insure, limited benefit package and weak management of the schemes, contributes to the low enrollment [4–6]. Moreover, socio-demographic characteristics like education levels, gender, household income, size of the family, marital status and location can determine the ability to enroll or re-enroll to the CHF/TIKA scheme [7–9]. In addition, social marketing remains to be a corner stone factor to the enrolment and re-enrolment to CHF/TIKA.

The health care financing in Tanzania comes from taxation (21%), voluntary pre-payment schemes (2%), social insurance schemes (3%), donor funding (48%), and OPP (25%) [10].

The Community Health Fund (CHF) program in Tanzania is a voluntary pre-payment scheme. It was established by the 2001 act, after a successful pilot in the Igunga district in 1996. The pilot was a result of health sector reforms in the health system financing structure, that is, from the provision of free health care services to the introduction of a cost-sharing policy in 1993. In 2009, ‘*Tiba kwa Kadi*’ (TIKA) was launched; its mode of operation is the same as CHF, except that its focus is on urban settings.

The Community Health Fund/Tiba Kwa Kadi (CHF/TIKA) membership is based on household enrollment, with a currently pre-defined household size of up to 6 members [11]. The membership is renewed every 12 months, and the annual contributions from each household are defined by the respective Local Government Authorities (LGAs), and vary across districts and mounts from Tanzanian shillings (Tsh) 5,000 -15,000 (i.e. between $3 – 9 USD/year) [12]. The CHF/TIKA schemes are also subsidized by the Government of Tanzania (GoT) through the National Health Insurance Fund (NHIF) in the form of matching grants (*Tele kwa Tele*), thereby complementing the members’ contributions with an amount equal to the premium decided by the Local Government Authority [11, 13]. Despite these initiatives to improve health services coverage through insurance schemes, the Tanzanian pre-payment schemes are still far from achieving the 30% coverage rate that was expected to be achieved by 2015 [10]. This might have been to several factors one of which is unorganized and fragmented social marketing approaches.

Moreover, the current national CHF/TIKA coverage remains very low at about 8% of the total population, with enormous regional variations [11]. This is entirely too low compared to the population of informal sector workers and their dependents, which represent more than 70% of the entire population [14, 15], this is also explained in by (Ministry of Health and Social Welfare, Tanzania Health Financing Strategy 2015-2025 Path towards Universal Health Coverage, 3rd Draft March 17th, 2015, unpublished). Existing evidence has documented the variations across districts in the manner in which CHF/TIKA is implemented and has attributed the variable performance of the schemes to the strategies used for implementation [11]. However, there is paucity of evidence on the role of social marketing strategies on CHF performance and its impact as a whole.

The Social Marketing discipline mainly relies on the principles and techniques developed by the commercial marketing sectors to influence consumer behavior. Social marketing may apply these to changing health behaviors, while emphasizing a set of communication or behavior change activities that are applied to market a product for a social good. It is characterized by a marketing mix approach, comprised of the 4Ps, namely product, price, place and promotion [16]. There are several social marketing approaches such as community sensitization, radio and football games all of which has 4 Ps. With community health insurance evolution in Tanzania and elsewhere, less investment has been made in the area of social marketing, especially in the CHF/TIKA schemes. The social marketing approach is an important tool, as it promotes demand creation. Social marketing has been demonstrated to have an impact on other interventions, such as, encouraging people to sleep under treated mosquito nets, discouraging people from excessive alcohol intake and cigarette smoking, and condom use promotion [17].

This study aimed to identify barriers and assessed facilitators to the social marketing strategies used to enroll and re-enroll people in CHF/TIKA schemes in two regions of Tanzania. We also explored the barriers to enrolling people in the schemes through the existing social marketing strategies in the study areas. Results from this study may inform programs and projects geared to increase the coverage of health insurance schemes and thus potentially increase health services to all people with varying economic status.

## Methods

### Study design

This cross-sectional study used quantitative methods to collect and analyze data. The study was conducted between November, 2014 and March 2015. Quantitative methods design was used as we intended to get quantifiable information related to enrollment and re-enrollment and their determinants.

### Study setting

The study was conducted in 2 regions of Tanzania representing the high (30%) and low (7%) performing regions with respect to CHF/TIKA enrollment levels as recorded by NHIF in 2015. Administratively, the Tanzania mainland has 26 regions. Only 5 regions are high performing and the rest are low performing [11]. The study was conducted in four districts within two regions of Tanzania: Singida (Singida Municipal Council and Iramba District Council) representing the high performing region and Shinyanga (Shinyanga Municipal Council and Shinyanga District Council) represents the low performing region. The CHF/TIKA enrollment rate of Singida was 33%, whereas that of Shinyanga was 7%. Availability of tracer medicine in Singida was 75%, while that of Shinyanga was 83% [18, 19].

### Sample size estimation

We used a random formula [20] with the proportion of the Tanzanian population enrolled in CHF/TIKA, which was 7.9% according to HPSS Project and NHIF reports [11]. Then, a calculation of the representative sample of the community, aged 18 years and above, was estimated considering a 95% confidence level or ά-error 0.05, marginal error of 3% and complex design effect of 1.5. Based on this calculation, the sample size was 466, which further increased by 5% (496) to account for a non-response rate.

### Data collection

We used a structured questionnaire (Additional file 1) containing five sections (general information, demographics, information about people who have enrolled in CHF/TIKA, information about people who have never enrolled in CHF/TIKA, and information about CHF/TIKA social marketing to obtain quantitative information.

### Sampling methods

A multi-stage sampling technique was used. In the first stage, regions were classified into high and low performing cohorts in terms of CHF/TIKA enrollment. The high performing regions were those with CHF/TIKA coverage of 7.9% and above, while the low performing regions were those with less 7.9%. Using a lottery method, random sampling was done to select regions with low and high performance. While in the second stage, there was a selection of four study districts, health facilities and community participants. From each selected region, one rural district and one urban district were selected, random sampling was employed to select the rural districts, among many, but there were only one urban district in each of the two regions and therefore selected by convenience. Then from each district 1 health center and 2 dispensaries were randomly selected from a cluster of health facilities in the respective districts.

A total of 12 public health facilities were randomly selected (six from each region) for this study, of which two were dispensaries and one was a health center. Only public health facilities were considered in this study because CHF/TIKA is not yet accepted in non-public health facilities in Tanzania. The interviewees were obtained by dividing a total sample of 496 by four (4) districts (i.e. - 124 participants per selected district).

Conveniently, at each dispensary and health center a sub-sample size of 25 and 74 respondents, respectively, were recruited for the study. Participants from the community were obtained based on their attendance/appearance at the 12 health care facilities that were selected randomly in the participating districts.

The exit interviews were conducted with participants after receiving health services at the health facility. The selection of study subjects was done by ensuring a balance of number of both CHF/TIKA and non-CHF/TIKA enrolled members.

### Data analysis

The collected quantitative data was assessed for completeness and consistency of information on a daily basis. Thereafter the data was coded and entered into the computer database using Epidata Version 3.1

Anonymous dual entry was performed as a means to ensure accuracy and correctness of the data entered and as a means of validation. Data was then exported to STATA version 12 (STATA Corp Inc., TX, USA) for data cleaning and analysis.

Descriptive statistics was used to summarize data, with proportions for categorical variables and means or medians with their respective measures of dispersion for continuous variables. The Chi - Square test (*χ* ^2^) was used to compare categorical data and Odds ratios (OR) with 95% Confidence Intervals (CI) were computed and used to determine the strength of association. Grouping income and family size into low/high and small/larger respectively was based on moment rules (central limit theorem approach) as it is elaborated by Bati et al (2013) and Tolossa et al (2014) [21, 22]. Multivariate logistic regression analysis was performed (education level, settings and culture) to get the strength of independent variables on the outcomes (dependent variable) of interest. Variables were entered in the logistic model if they had a value of *p* ≤ 0.2 in the univariate analysis [23]. Crude and Adjusted Odds Ratios (AOR), with 95% confidence intervals (CI), were reported. Associations and differences between variables were considered statistically significant if *p* < .05. Associations and differences were considered statistically significant if *p* < .05. Dependent variables for this study was readiness to enroll or to re - enroll in the CHF/TIKA scheme, while the independent variables included social and demographic variables (age, sex, family size, level of education, occupation, income level and marital status), individual residential setting (urban and rural districts), number of social marketing Strategies ever exposed to and enabling factors (influence of leaders, social, economic, by-laws, act, e.t.c). Influence of leaders here is defined as readiness of political, religious and civic leaders to advocate the scheme during meetings.

## Results

### Socio-demographic characteristics

A total of 496 people aged 18 years and above were recruited, while 460 (92.7%) consented to participate in this study. Of those who participated in the study more than half (51.1%) were from rural settings. The mean age, with standard deviations in parentheses, of the respondents was 34.2(11.8) years. Females accounted for about 57% of the respondents. One third (33.0%) of the respondents were aged 25–34 years, and more than three quarters (77.3%) were married.

More than two-thirds (69.5%) of the respondents had a primary level of education, and about 62% were peasants/farmers. The majority (87.8%) of the respondents reported that heads of their households were males.

The mean household monthly income and family size were Tshs.199, 884.35 (348,272.5), equivalent to 98.95 (170.72 USD), and 6.0 (2.8) members [Table 1].

**Table 1:** Socio-demographic of the respondents by enrolment status

Characteristics	Total, *n* (%)	Shinyanga, *n* (%)	Singida, *n* (%)
Response rate	460(92.7)	228(91.9)	232(93.5)
Residential Locality			
Rural	235(51.1)	116(50.9)	119(51.3)
Urban	225(48.9)	112(49.1)	113(48.7)
Level of Health Facility			
Dispensary	202(43.9)	103(45.2)	99(42.7)
Health Centre	258(56.1)	125(54.8)	133(57.3)
Status of enrollment in CHF/TIKA			
CHF/TIKA Member	229(49.8)	115(50.4)	114(49.1)
Non CHF/TIKA Member	231(50.2)	113(49.6)	118(50.9)
Sex			
Male	200(43.5)	104(45.6)	96(41.4)
Female	260(56.5)	124(54.4)	136(58.6)
Age Group			
< 25	109(23.7)	50(21.9)	59(25.4)
25-34	152(33.0)	65(28.5)	87(37.5)
35-44	114(24.8)	69(30.3)	45(19.4)
45-54	52(11.3)	24(10.5)	28(12.1)
55+	33(7.2)	20(8.8)	13(5.6)
Marital Status			
Married	356(77.3)	176(77.2)	180(77.6)
Single	55(12.0)	19((8.3)	36(15.5)
Others	49(10.7)	33(14.5)	16(6.9)
Education level			
No formal education	57(12.4)	40(17.5)	17(7.3)
Primary	320(69.5)	148(64.9)	172(74.2)
Secondary	78(17.0)	38(16.7)	40(17.2)
Others	5(1.1)	2(0.9)	3(1.3)
Occupation			
Farmer/peasants	287(62.4)	141(61.8)	146(62.9)
Employed	16(3.5)	7(3.1)	9(3.9)
Business	86(18.7)	43(18.9)	43(18.5)
Student	14(3.0)	5(2.2)	9(3.9)
None	23(5.0)	13(5.7)	10(4.3)
Others	34(7.4)	19(8.3)	15(6.5)
Sex of Head of Household			
Male	404(87.8)	196(86.0)	208(89.7)
Female	56(12.2)	32(14.0)	24(10.3)
Mean monthly income	199,884.4	215,154.2	185,489.0
Mean household size	6.0	6.6	5.3

### Social marketing strategies used in enrolling people to community health funds (CHF/TIKA)

A total of 418/460 (90.9%) of the respondents reported having heard CHF/TIKA educational messages. Of those who had no such educational messages 24(57.1%) were from low enrollment areas, and all were not yet enrolled. Sensitization messages at the health facility via health service providers 206(90.0%), local radio media 126(55%), and influence of community leaders 124(54.1%) were the common marketing strategies reported by CHF/TIKA members in both low and high enrollment sites. The use of mosques or churches as part of the CHF/TIKA social marketing approach was significantly (24.6% vs 13.0% *p* = 0.019) as reported by the site with a high enrollment rate compared to the low enrollment rate site (Tables 2 and 3). On the other hand, out of 229 CHF members, 184(80.3%) members reported that they joined the scheme after being sensitized at the health facilities.

**Table 2:** Social marketing strategies reported by CHF/TIKA enrolled clients

Strategy	Total, *n* (%)	Low (Shinyanga), *n* (%)	High (Singida), *n* (%)	*P*-Value
Traditional Dances	4(1.7)	3(2.6)	1(0.9)	0.314
Football games	2(0.9)	2(1.7)	0(0.0)	0.251
Sensitization at the health facility via health service providers	206(90.0)	100(87.0)	106(93.0)	0.097
Radio media (local)	126(55.0)	60(52.2)	66(57.9)	0.231
Radio media (national)	103(45.0)	48(41.7)	55(48.2)	0.196
TV (national)	45(19.7)	19(16.5)	26(22.8)	0.151
TV (local)	39(17.0)	16(13.9)	23(20.2)	0.139
News papers	15(6.6)	9(7.8)	6(5.3)	0.304
Influence of community leaders via meetings	124(54.1)	59(51.3)	65(57.0)	0.231
Mosque or Church	43(18.8)	15(13.0)	28(24.6)	0.019
Mixed approaches	3(1.3)	3(2.6)	0(0.0)	0.125
House to house sensitizations	31(13.5)	17(14.8)	14(12.3)	0.360
Banners	36(15.7)	16(13.9)	20(17.5)	0.283
Others(relatives, schools, brochures etc)	22(9.6)	16(13.9)	6(5.3)	0.022

**Table 3:** Social marketing strategies reported by non-enrolled clients on CHF/TIKA

Strategy	Total *n* (%)	Low (Shinyanga), *n* (%)	High (Singida), (%)	*P*-Value
Traditional Dances	3(1.6)	1(1.1)	2(2.0)	0.544
Football games	5(2.6)	1(1.1)	4(4.0)	0.223
Sensitization at the health facility via health service providers	129(68.3)	50(56.2)	79(79.0)	0.001
Competition	2(1.1)	0(0.0)	2(0.0)	0.279
Radio media (local)	108(57.1)	49(55.1)	59(59.0)	0.345
Radio media (national)	98(51.9)	45(50.6)	53(53.0)	0.425
TV (national)	35(18.5)	17(19.1)	18(18.0)	0.496
TV (local)	31(16.4)	15(16.9)	16(16.0)	0.514
News paper	7(3.7)	5(5.6)	2(2.0)	0.177
Influence of community leaders via meetings	74(39.2)	23(25.8)	51(51.0)	*p* < 0.0001
Mosque or Church	32(16.9)	8(9.0)	24(24.0)	0.005
Mixed approaches	6(3.2)	3(3.4)	3(3.0)	0.602
House to house sensitizations	22(11.6)	11(12.4)	11(11.0)	0.473
Burners	22(7.9)	7(7.9)	15(15.0)	0.096
Others(relative, schools, brochures etc)	26(13.8)	17(19.1)	9(9.0)	0.036

The use of a community sensitization strategy at the health facility via health service providers (79.0% vs 56.2%, *p* = 0.001), influence of community leaders via meetings (51.0% vs 25.8%, *p* < 0.0001) and mosques or churches (24.0% vs 9.0%, *p* = 0.005) as social marketing strategies for CHF/TIKA were significantly reported by non CHF/TIKA members from study sites with high enrollment rates as compared with sites having low enrolment rates

### Determinants for enrolling or re-enrolling with CHF

A total of 376(81.7%) participants were ready to enroll or to re-enroll with CHF scheme. Out of 10; six factors namely locality, family monthly average income, sex, marital status, education level and family size were significant during univariate analysis. However, during multivariate analysis only two factors namely marital status and family size were the significant factors which were associated joining or re-joining to the CHF/TIKA scheme (Table 4). Married, divorced, widow, separated respondents were two times more likely to be ready to enroll or re-enroll with the scheme as compared to single respondents. Respondents from household with household with six and above members were about two times more likely to report that they are ready to enroll or re-enroll the scheme as compared to those from families with five and less household members.

**Table 4:** Determinants for enrollment or re-enrollment into CHF/TIKA scheme

Determinant	Ready to enroll/re-enroll, *n* (%)	*P*-Value	COR	AOR
Region CHF enrolment rate				
Low	186(81.6)	0.513	1	
High	190(81.9)		1.0(0.6-1.6)	
Locality				
Rural	185(78.7)	0.056	1	1
Urban	191(84.9)		1.5(0.9-2.5)^a^	1.5(0.9-2.4)
Sex				
Male	157(78.5)	0.073	1	1
Female	219(58.2)		1.5(0.9-2.4)^a^	1.4(0.8-2.2)
Age group				
< 35 years	218(83.5)	0.155	1	
35+ years	158(79.4)		0.8(0.5-1.2)	
Marital status				
Single	40(72.7)	0.056	1	1
Married, divorced, widow etc	336(83.0)		1.8(1.0-3.5)^a^	2.0(1.1-3.8)
Education				
None/primary	310(82.2)	0.330	1	1
Secondary/above	66(79.5)		0.84(0.5-1.5)	
Family monthly average income				
Low (< median)	170(82.5)	0.507	1	
High (≥median)	193(82.1)		1.0(0.6-1.6)	
Family size (number of members)				
Small (<median 6)	174(78.7)	0.061	1	1
Larger(≥median 6)	200(84.7)		1.5(0.9-2.4)^a^	1.5(1.0-2.5)
Number of marketing strategies ever exposed				
Never/one strategy	113(83.1)	0.366	1	
Two & more strategies	263(81.2)		0.9(0.5-1.5)	
Influence of leaders ever heard CHF message community leaders				
No	175(79.5)	0.126	1	
Yes	167(84.3)		1.4(0.8-2.4)	

^a^Significant factors during bivariate analysis at *p* ≤ 0.2

## Discussion

This study aimed at assessing the influence of marketing strategies and socio-demographic and social on enrollment and re-enrollment in the Community Health Fund/Tiba Kwa Kadi (CHF/TIKA) in Tanzania.

We found that there are several marketing strategies implemented in the study area, the strategies ranged from facility base sensitization, use of mainstream media to community based communication strategies. Although majority (91%) of the participants were aware of the CHF/TIKA, detailed information about CHF/TIKA and enrolment rate in the scheme were low. Socio-demographic characteristics were important in decisions to enroll or re-enroll.

While social marketing in the field of community health fund is a new approach, it has been used extensively in international health interventions for instance contraceptives and oral rehydration therapy, insecticide-treated mosquito nets, and intermittent preventive treatment for Malaria with Sulphadoxine – pyrimethamine. Additionally, social marketing is being used with more frequency for a diversity of health-related topics to achieve sustained changes in healthy behaviors [17, 24, 25].

In the context of a community health fund, social marketing approaches can be used to get to know the beneficiary, as it involves active participation.

This in turn will build awareness among the primary target audience and secondary target audience in their decision to join community health funds, as well as a possibility for behavior change.

In this study it was found that majority (91%) of the participants were aware with CHF/TIKA scheme. Important sources of information were health service providers, mass media and community leaders. However, a majority of those who were aware had limited detailed information about CHF/TIKA, which may be due to low utilization of other available types of social marketing strategies including; traditional dances, football games, local and national television and news papers.

Our argument is also supported by Sheng Chung Lo [15], who documented that high knowledge levels of the service users about the service reduces the chance of losing the customer and minimizes negative attitudes to the service delivered. This implies that there is a need for more effectively utilizing the above strategies for increasing and sustaining CHF/TIKA enrolment rates [15].

In this study, a significant contribution to enrollment rates was attributed to the influence of community leaders. This point to the importance of involving such leaders, in view of enrolling more people in CHF/TIKA, leading to increased financial and risk pooling. Enrolling more people is of paramount importance and this should be done during a specific period (i.e. during crop harvesting time), especially for the rural settings. In addition, enrollment of members should be done before a beneficiary is sick, since it was noted that a majority of CHF/TIKA members were sick at the time of registration that is they said they joined after being sensitized by health service providers at the health facilities. This may precipitate the utilization of the entire existing fund at once, implying little risk pooling and an adverse selection problem.

In this study social marketing by health service providers was not significantly associated with an increase in CHF/TIKA enrollment. This was also the case for the study that was done in Tanzania by Borghi et al (2014), which was looking into the administrative cost of community-based health insurance [26].

The findings from various studies have revealed that households and community characteristics, such as age of the head of the household, gender and income were among the factors determining households’ membership in the micro-health insurance schemes in rural areas [27, 28]. In this study, the two socio-demographic characteristics of study respondents namely:-marital status and family size were the significantly associated enrollment or re-enrollment to the CHF/TIKA scheme. These findings are in line with two studies study done in Ghana which revealed marital status and level of education to be amongst influencing factors to enroll or to renew insurance membership [7, 8].

While in another study which was done in Ethiopia, it was revealed that; size of the family was positively associated with households’ decisions to willingly enroll into the scheme. As the number of the household members increase, the probability of willingness to enroll increased by 69% [9]. Such practice also has a consequence on the scheme since it affect the risk pooling principle as those who join have large family size thus increased chance of exhausting of all resource from the members within a short time.

### Limitations of the study

This study is limited in the sense that it depends on verbal responses and might therefore have included some misinformation due to recall bias. In dealing with this type of bias, we gave an opportunity for participants to use reference materials and also arranged questions in the order of providing opportunity to recall. Participants were those who presented at the health facilities and might not represent the true picture of the general population. However, we balanced the number of members and non-members of CHF/TIKA to ensure representation of all community members.

## Conclusion

This study identified several social marketing strategies employed to attract community members to enroll in the CHF/TIKA. The following strategies are among the ones identified; sensitization on CHF/TIKA in health facilities, use of mainstream media (mainly radio, Television and news papers), advocacy by leaders (political, civic and religious), traditional dances, sports and games. Majority of respondents had heard of CHF/TIKA; however, most members were enrolled after getting sick, implying that sensitization at the health facility via health service providers was the common social marketing strategy. It was also revealed that marital status and number of family members were the factors which influenced people to enroll the scheme. The available social marketing strategies are facing some limitations to attract many people to join the scheme as they only focus on provision of knowledge about the scheme and little is done to empower the community members so that they can influence on availability of medical commodities in health facilities and improve their own income to enhance affordability of the CHF/TIKA premiums.

There is a need of re-orienting social marketing strategies in order to address issues of equity in provision of information (not focusing in health facilities only), employ strategies that empower the communities to get detailed CHF/TIKA knowledge so as to reduces the chance of losing the customer and minimizes negative attitudes which is the driving factors for sustaining the scheme. Interventions for increasing enrolment rate to the CHF/TIKA scheme should also consider impending socio-demographic parameters such as family size and marital status..

## Additional file

Additional file 1: Questionnaire for Community Members. (DOC 161 kb)

## Abbreviations

BRN: Big results now
CHF/TIKA: Community health fund
DC: District council
DHSB: District health service board
DMO: District medical officer
FGD: Focus group discussion
HFGC: Health facility governing committee
LGA: Local government authority
MoHCDGEC: Ministry of health, community development, gender, elderly and children
NBS: National bureau of statistics
NHIF: National health insurance fund
NIMR: National institute for medical research
OOP: Out of pocket
PORALG: President's Office -Regional Administration and Local Government
WHO: World Health Organization

## References

[1] WHO, 2015: Out-of-pocket expenditure on health as a percentage of private expenditure on health Situation and trends as at. http://apps.who.int/iris/bitstream/10665/170250/1/9789240694439_eng.pdf. Accessed 20 Aug 2015.

[2] Ranson MK (2002). Reduction of catastrophic health care expenditures by a community-based health insurance scheme in Gujarat, India: current experiences and challenges. Bull World Health Organ.

[3] Spreeuwers AM, Dinant GJ. Success and failure in social health insurance in Sub-Saharan Africa: what lessons can be learnt? 2011. at;- http://afhea.org/en/2014-02-11-15-47-44/research-dissemination/2014-07-22-11-43-33/33-health-insurance/1085-success-and-failure-in-social-health-insurance-in-sub-saharan-africa-what-lessons-can-be-learnt. Accessed 2014.

[4] The national health insurance fund act, 1999 arrangement of sections. (1999).

[5] Kamuzora P, Gilson L (2007). Factors influencing implementation of the Community Health Fund in Tanzania. Health Policy and Planning.

[6] Jane M, August K, Suzan M, Gemini M, Josehine B. Determinants of Community Health Fund membership in Tanzania: a mixed methods analysis. BMC Health Services Research. 2014;14:538. doi:10.1186/s12913-014-0538-9. https://bmchealthservres.biomedcentral.com/articles/10.1186/s12913-014-0538-9. 10.1186/s12913-014-0538-9 PMC4246628 25411021

[7] Appiah SCY. The influence of socio-demographic characteristics on health care access among health insurance subscribers in Ghana. Edorium J Public Health 2015;2:1–10. www.edoriumjournalofpublichealth.com. http://www.ejpublichealth.edoriumjournals.com/archive/2015-archive/100003P16SA2015-appiah/100003P16SA2015-appiah.pdf.

[8] Boateng D, Awunyor-Vitor D. Health insurance in Ghana: Evaluation of policy holders’ perceptions and factors influencing policy renewal in the Volta region. Int J Equity in Health. The official journal of the International Society for Equity in Health. 2013;12:50. doi:10.1186/1475-9276-12-50. https://equityhealthj.biomedcentral.com/articles/10.1186/1475-9276-12-50. 10.1186/1475-9276-12-50 PMC3716631 23822579

[9] Melaku H, Shimeles O, Berhane M. Willingness to join community-based health insurance among rural households of Debub Bench District, Bench Maji Zone, Southwest Ethiopia. BMC Public Health. 2014;14:591. doi:10.1186/1471-2458-14-591. https://bmcpublichealth.biomedcentral.com/articles/10.1186/1471-2458-14-591. 10.1186/1471-2458-14-591 PMC4074337 24920538

[10] Ministry of Health and Social Welfare. The National Health Account Reports. 2012.

[11] Humba E (2011). Pioneering social health insurance in Tanzania: The case of the National Health Insurance Fund (NHIF). Improving Access through Effective Health Financing.

[12] Macha J, Harris B, Garshony B, Afaguba JE, Akasili J, Kuwawenuruwa A (2012). Borghi J; Factors influencing the burden of health care financing and the distribution of health care benefits in Ghana, Tanzania and South Africa. Health Policy Plan.

[13] Ministry of Health and Social Welfare: Tanzania Health Sector Strategic Plan IV 2015 – 2020. 2015. http://www.tzdpg.or.tz/fileadmin/documents/dpg_internal/dpg_working_groups_clusters/cluster_2/health/Key_Sector_Documents/Induction_Pack/Final_HSSP_IV_Vs1.0_260815.pdf.

[14] Ministry of Health Tanzania. Health Insurance Regulatory Framework Review. 2012. It was accessed in 2014, http://www.thdr.or.tz/docs/THDR-BP-8.pdf.

[15] Lo SC (2012). A Study of Relationship Marketing on Customer Satisfaction. Journal of Social Sciences.

[16] Arcaro P, Mannocci A, Saulle R, Miccoli S, Marzuillo C, La Torre G (2013). [Social marketing and public health]. Marketing sociale e sanità pubblica doi:10.7416/ai.2013.1927. Retrieved from http://www.seu-roma.it/riviste/annali_igiene/apps/autos.php?id=859. 10.7416/ai.2013.1927 23598808

[17] Hanson K, Kikumbih N (2003). Cost-effectiveness of social marketing of insecticide-treated nets for malaria control in the United Republic of Tanzania. Bulletin of the World Health Organization.

[18] Katherine E. Bliss, Cathryn Streifel. Targeting Big Results in Maternal, Neonatal, and Child Health. A trip report of the Center For Strategic and International Studies (CSIS) delegation to the United Republic of Tanzania; 2015.

[19] National Bureau of Statistics, Ministry of Finance, The United Republic of Tanzania - Mortality and Health and July, 2015 and Office of Chief Government Statistician Ministry of State, President Office, State House and Good Governance. It was accessed in 2015, http://www.nbs.go.tz/nbs/takwimu/census2012/Mortality_and_Health_Monograph.pdf.

[20] William_G._Cochran_Sampling_Techniques_Third_EdBookFi.org_.pdf. (1977). It was accessed in 2014, http://hbanaszak.mjr.uw.edu.pl/TempTxt/Cochran_1977_Sampling_Techniques__Third_Edition.pdf.

[21] Bati J, Legesse M, Medhin G (2013). Community's knowledge, attitudes and practices about tuberculosis in Itang Special District, Gambella Region, South Western Ethiopia. BMC Public Health.

[22] Tolossa D, Medhin G, Legesse M (2014). Community knowledge, attitude and practices towards tuberculosis in Shinile town, Somali regional state, eastern Ethiopia: a cross-sectional study. BMC Public Health.

[23] Mitchell H. Katz. Multivariate Analysis: A practical Guide for Clinicians, Second addition. 2010.

[24] Sabine Gies, Sheick Oumar Coulibaly, Florence Tiemegna Ouattara, Clotilde Kay, Bernard John Brabin5 and Umberto Dalessandro. A community effectiveness trial of strategies promoting intermittent preventive treatment with sulphadoxine-pyrimethamine in pregnant women in rural Burkina Faso. 2008. 10.1186/1475-2875-7-180 PMC2563022 18801158

[25] Joanna RM Armstrong Schellenberg, Salim Abdulla, Rose Nathan et al. Effect of large-scale social marketing of insecticide-treated nets on child survival in rural Tanzania. The Lancent. 2001;357(9264):1241–47. http://dx.doi.org/10.1016/S0140-6736(00)04404-4. Accessed 21 Apr 2001. 10.1016/S0140-6736(00)04404-4 11418148

[26] Borghi J, Maluka S, Kuwawenaruwa A, Makawia S, Tantau J, Mtei G, Ally M, Macha J (2013). Promoting universal financial protection: a case study of new management of community health insurance in Tanzania. Health Res Policy Syst.

[27] Bruno Meessen, Universal Health Coverage: the work of urban planners, not architects. Health Financing in Africa Le blog. 2014. It was accessed in 2014, can be accessed at http://www.healthfinancingafrica.org/home/universal-health-coverage-the-work-of-urban-planners-not-architects.

[28] Lammers J. and Warmadam S. Adverse selection in Voluntary Micro Health Insurance in Nigeria, AIDS Research Series;2010.10-06

