# Supplementary material for: Barriers and facilitators to enrollment and re-enrollment into the community health funds/Tiba Kwa Kadi (CHF/TIKA) in Tanzania: a cross-sectional inquiry on the effects of socio-demographic factors and social marketing strategies
Source: BMC Health Serv Res. 2017 Apr 27;17:308. doi: 10.1186/s12913-017-2250-z (PMC5408418; doi:10.1186/s12913-017-2250-z)
Supplement: Additional file 1: — Questionnaire for Community Members. (DOC 161 kb) [file 12913_2017_2250_MOESM1_ESM.doc]

# Supplementary File 1: Questionnaire for Community Members

# BARRIERS AND FACILITATORS TO ENROLLMENT AND RE-ENROLLMENT INTO THE COMMUNITY HEALTH FUNDS/TIBA KWA KADI (CHF/TIKA) IN TANZANIA

| **QUESTIONNAIRE FOR COMMUNITY MEMBERS** |
| --- |

# Questionnaire number_______________

**SECTION 1**: - GENERAL INFORMATIONS

| No | Question | Response | Code |
| --- | --- | --- | --- |
| 1 | Date of an Interview | |__|__|/|__|__|/|__|__||__|__| (dd/mm/yyyy) |  |
| 2 | The name of the interviewer |  |  |
| 3 | Region | 1. Shinyanga 2. Singida |  |
| 4 | District | 1.Shinyanga Municipal Council 2.Shinyanga District Council 3. Singida Municipal Council 4. Ilamba District Council |  |
| 5 | Type of District | 1.Rural 2. Urban |  |
| 6 | The Level of Health Facility | 1.Dispensary 2. Health Center |  |
| 7 | For How long (years) have you been living in this District? | |____|____| |  |

**SECTION 2: - SOCIO-DEMOGRAPHIC** INFORMATION

| No | Question | Response | Code |
| --- | --- | --- | --- |
| 1 | SEX | 1. Male 2. Female |  |
| 2 | a)Year of birth | |_____|_____||_____|_____| |  |
|  | b) What is your age? | |_____|_____| |  |
| 3 | Marital status? | 1. Married 2. Single 3. Divorced 4. Widow 5. Separated 6. Cohabiting |  |
| 4 | Level of education? | 1. None 2. Adult education 3. Primary school education 4. Secondary school education5. certificate 6. Diploma7. university degree or more |  |
| 5 | What is your average income earned per month? | a)      Daily (Tshs)_________ |  |
|  |  | b)      Weekly (Tshs)_________ |  |
|  |  | c)      Monthly (Tshs)_________ |  |
| 6 | How many are you in your household? _________ |  |  |

**SECTION 3: INFORMATION ON THE PEOPLE WHO HAVE ENROLLED IN CHF/TIKA**

| No | Question | Response | Codes |
| --- | --- | --- | --- |
| 1 | Which scheme have you been enrolled? | 1.CHF 2.TIKA |  |
| 2 | For how long have you been in the CHF/TIKA scheme? | Year |_____|_____|Month |_____|_____| |  |
| 3 | What motivated you to join CHF? |  |  |
|  | a) I wanted to have an insurance to protect me | 1.Yes 2.No |  |
|  | b) I was sensitized by community leaders | 1.Yes 2.No |  |
|  | c) Sensitization from health service providers | 1.Yes 2.No |  |
|  | d) Sensitization from my neighbours and friends | 1.Yes 2.No |  |
|  | f) Sensitization from other sources (Please mention)__________________________________ |  |  |
| 4a. | Who paid for your CHF premium? | 1.My self 2.My Husband/Wife 3.CBO/NGO 4.District Council 5.Others |  |
| 4b. | If others paid for your premium (Please mention)__________________________________________ |  |  |
| 5 | Is your premium paid through money or other things? | 1. Things 2 .Money |  |
| 6 | If through money, which modality did you use to pay your premium? | 1.Once |  |
|  |  | 2. By Installment |  |
| 7 | How much do you pay (Tsh) per year? |  |  |
| 8 | If are other things, what are they? |  |  |
|  | a) Hen, Goat | 1.Yes 2.No |  |
|  | b) Cereals | 1.Yes 2.No |  |
|  | c)Others, mention | 1.Yes 2.No |  |
| 9 | How many in monetary terms if you convert those things? (Tshs) | ________________ |  |
| 10 | Do you feel what you are contributing is enough per year? | 1.Yes 2.No |  |
| 11 | If is NO fro Qn 10 what is your suggestions or the premium? | ______________ |  |
| 12 | For the quality service you are getting now are you ready to re-enroll (re-pay) next year? | 1. Yes |  |
|  |  | 2. No→ Qn 14 |  |
| 13 | If YES to Qn 12 why are ready to continue paying for your premium? |  |  |
|  | a) Medicine availability throughout the year | 1.Yes 2.No |  |
|  | b) Good Customer care from health service providers | 1.Yes 2.No |  |
|  | c) Getting health services any time when it is needed | 1.Yes 2.No |  |
|  | d) Others (Mention)_________________________________________________ |  |  |
| 14 | During which period of the year will you be like to be enrolling for CHF/TIKA? | |  ­­­­­­­­___| years |  |
| 15 | If it is a NO answer to Qn 12 why are you not ready to re-enroll again? |  |  |
|  | a) Bad languages by the service providers | 1.Yes 2.No |  |
|  | b) Not getting feedback of our complaints | 1.Yes 2.No |  |
|  | c) Medicine stock outs | 1.Yes 2.No |  |
|  | d) Bureaucracy in getting health services | 1.Yes 2.No |  |
|  | e) Low income earned in our family | 1.Yes 2.No |  |
|  | f) High CHF premium per year | 1.Yes 2.No |  |
|  | g) Out of pocket payment is smaller than the annual CHF premium | 1.Yes 2.No |  |
|  | h) I do not understand well the CHF/TIKA | 1.Yes 2.No |  |
|  | i) CHF services does not cross facility/ district boarders | 1.Yes 2.No |  |
|  | j) CHF services does not offer referral linkage | 1.Yes 2.No |  |
|  | k) Few services /Low minimum benefit package to the members | 1.Yes 2.No |  |
|  | l) In my family majority have been exempted from cost sharing (i.e. Under 5, elderly) | 1.Yes 2.No |  |
|  | m)Others (Mention)______________________________ |  |  |

**SECTION 4**: INFORMATION FOR THOSE WHO HAVE NEVER ENROLLED INTO CHF/TIKA

| 1 | Why have you not ever enrolled for CHF? |  |  |
| --- | --- | --- | --- |
|  | a)      I heard that services are not satisfactory | 1.Yes 2.No |  |
|  | b)      There is no sufficient medicine at the facilities | 1.Yes 2.No |  |
|  | c)      Small financial income | 1.Yes 2.No |  |
|  | d)     High annual CHF premium fee | 1.Yes 2.No |  |
|  | e)      Out of Pocket money is small as compared to the CHF premium | 1.Yes 2.No |  |
|  | f)       Communities do not have a very good understanding of the CHF | 1.Yes 2.No |  |
|  | g)      CHF services does not cross facility/ district boarders | 1.Yes 2.No |  |
|  | h)      CHF services does not offer referral linkage | 1.Yes 2.No |  |
|  | i)        Few services/low minimum benefit package to members | 1.Yes 2.No |  |
|  | j)        In my family majority have been exempted from cost sharing (i.e. Under 5, elderly) | 1.Yes 2.No |  |
|  | k)      Others (mention)________ |  |  |
| 2 | Are you ready to join/enroll for CHF right now? | 1.Yes 2.No |  |
| 3 | If NO to Qn 2 Why? |  |  |
|  | a)      I heard that services are not satisfactory | 1.Yes 2.No |  |
|  | b)      There is no sufficient medicine at the facilities |  |  |
|  | c)      Small financial income |  |  |
|  | d)     High annual CHF premium fee |  |  |
|  | e)      Out of Pocket money is small as compared to the CHF premium |  |  |
|  | f)       Communities do not have a very good understanding of the CHF | 1.Yes 2.No |  |
|  | g)      CHF services does not cross facility/ district boarders | 1.Yes 2.No |  |
|  | h)      CHF services does not offer referral linkage | 1.Yes 2.No |  |
|  | i)        Few services/low minimum benefit package to members | 1.Yes 2.No |  |
|  | j)        In my family majority have been exempted from cost sharing (i.e. Under 5, elderly) | 1.Yes 2.No |  |
|  | k)      Others (mention)________ |  |  |
| 4 | If yes to Qn 2 why? |  |  |
|  | a) availability of medicine throughout the year | 1.Yes 2.No |  |
|  | b) To get good customer care from health services provider | 1.Yes 2.No |  |
|  | c) It helps in getting services whenever we need it. | 1.Yes 2.No |  |
|  | Others (mention)___________________________________ |  |  |
|  |  |  |  |
| 5 | Is your premium paid through money or other things? | 1. Money 2. Other things |  |
|  | If through money, which modality did you use to pay your premium? |  |  |
| 6 | Is your premium paid through money or other things? | 1.Once |  |
|  | If through money, which modality did you use to pay your premium? |  |  |
|  |  | 2. By installment |  |
| 7 | If are other things, what are they? | 1.Yes 2.No |  |
|  | a) Hen, Goat |  |  |
|  | b) Cereals |  |  |
|  | c)Others, mention |  |  |

**SECTION 5:** INFORMATION ON CHF SOCIAL MARKETING

| No | Question | Response | Code |
| --- | --- | --- | --- |
| 1 | Have you ever heard in this community anything regarding enrollment to CHF/TIKA? | 1.Yes 2.No |  |
| 2 | Which social marketing strategies are used in sensitizing communities to join CHF? (Circle as many option as you can_ |  |  |
|  | a)      Traditional dances | 1.Yes 2.No |  |
|  | b)      Football match | 1.Yes 2.No |  |
|  | c)      Different competition | 1.Yes 2.No |  |
|  | d)     National Radio | 1.Yes 2.No |  |
|  | e)      Local Radio | 1.Yes 2.No |  |
|  | f)       National TV | 1.Yes 2.No |  |
|  | g)      Local TV | 1.Yes 2.No |  |
|  | h)      News paper | 1.Yes 2.No |  |
|  | i)        Influence from community and political leaders or voluntary | 1.Yes 2.No |  |
|  | j)        Mosque or church | 1.Yes 2.No |  |
|  | k)      Mixed methods | 1.Yes 2.No |  |
|  | Others (mention)________ |  |  |
| 3 | If you are a new or old member of CHF which way did motivate you to take up decision to join CHF/TIKA? (Circle only one option) |  |  |
|  | a)      Traditional dances |  |  |
|  | b)      Football match |  |  |
|  | c)      Different competition |  |  |
|  | d)     National Radio |  |  |
|  | e)      Local Radio |  |  |
|  | f)       National TV |  |  |
|  | g)      Local TV |  |  |
|  | h)      News paper |  |  |
|  | i)        Influence from community and political leaders or voluntary |  |  |
|  | j)        Mosque or church |  |  |
|  | k)      Mixed methods |  |  |
|  | Others (mention)________ |  |  |
| 4 | Do you know that; the Government is providing a matching grant when you have enrolled for CHF/TIKA? | 1.Yes 2.No |  |
| 5 | What do you think are main obstacles/barriers to successful CHF/TIKA enrollment? | 1.Yes 2.No |  |
|  | a)Medicine shortages/stock outs | 1.Yes 2.No |  |
|  | b)Inadequate human resource for health | 1.Yes 2.No |  |
|  | c)low minium benefit packages | 1.Yes 2.No |  |
|  | e) Long waiting time | 1.Yes 2.No |  |
| 6 | What do you think are social marketing strategies which can sensitize more people to join CHF/TIKA? |  |  |
|  | a) Traditional Dance | 1.Yes 2.No |  |
|  | b) Football | 1.Yes 2.No |  |
|  | c) Competition | 1.Yes 2.No |  |
|  | d) National Radios | 1.Yes 2.No |  |
|  | e) Local Radios | 1.Yes 2.No |  |
|  | f) National TV | 1.Yes 2.No |  |
|  | g) Local TV | 1.Yes 2.No |  |
|  | h) Newspaper | 1.Yes 2.No |  |
|  | i) Influence from community and political leaders, voluntary | 1.Yes 2.No |  |
|  | j) Mosque/ Church | 1.Yes 2.No |  |
|  | k)Mixed methods | 1.Yes 2.No |  |
|  | l)Others (mention)________________________________ |  |  |
| 7 | What do you advice to make the CHF/TIKA more appealing? |  |  |
|  | a)Medicine availability throughout the year | 1.Yes 2.No |  |
|  | b)To reduce the CHF premium | 1.Yes 2.No |  |
|  | c)Referral system should be up (include) to the regional referral Hospital | 1.Yes 2.No |  |
|  | d) Re enrollement should be done after every three years. | 1.Yes 2.No |  |
|  | a) Others (mention)___________ |  |  |

**THANK YOU VERY MUCH FOR YOUR COOPERATION**
